# Development of an Algorithm to Identify Patients with Physician-Documented Insomnia

**DOI:** 10.1038/s41598-018-25312-z

**Published:** 2018-05-18

**Authors:** Uri Kartoun, Rahul Aggarwal, Andrew L. Beam, Jennifer K. Pai, Arnaub K. Chatterjee, Timothy P. Fitzgerald, Isaac S. Kohane, Stanley Y. Shaw

**Affiliations:** 1 Center for Systems Biology; Center for Assessment Technology & Continuous Health (CATCH), Massachusetts General Hospital, Boston, MA USA; 2 Harvard Medical School, Boston, MA USA; 3 Department of Biomedical Informatics, Harvard Medical School, Boston, MA USA; 4 Merck & Co., Inc., Boston, MA USA; 5 Present Address: McKinsey & Company, Boston, MA USA; 6 Merck & Co., Inc., West Point, PA USA; 7 Present Address: One Brave Idea, Division of Cardiovascular Medicine, Brigham and Women’s Hospital, Boston, MA USA; 8 Present Address: Center for Computational Health, IBM Research, Cambridge, MA USA

## Abstract

We developed an insomnia classification algorithm by interrogating an electronic medical records (EMR) database of 314,292 patients. The patients received care at Massachusetts General Hospital (MGH), Brigham and Women’s Hospital (BWH), or both, between 1992 and 2010. Our algorithm combined structured variables (such as International Classification of Diseases 9th Revision [ICD-9] codes, prescriptions, laboratory observations) and unstructured variables (such as text mentions of sleep and psychiatric disorders in clinical narrative notes). The highest classification performance of our algorithm was achieved when it included a combination of structured variables (billing codes for insomnia, common psychiatric conditions, and joint disorders) and unstructured variables (sleep disorders and psychiatric disorders). Our algorithm had superior performance in identifying insomnia patients compared to billing codes alone (area under the receiver operating characteristic curve [AUROC] = 0.83 vs. 0.55 with 95% confidence intervals [CI] of 0.76–0.90 and 0.51–0.58, respectively). When applied to the 314,292-patient population, our algorithm classified 36,810 of the patients with insomnia, of which less than 17% had a billing code for insomnia. In conclusion, an insomnia classification algorithm that incorporates clinical notes is superior to one based solely on billing codes. Compared to traditional methods, our study demonstrates that a classification algorithm that incorporates physician notes can more accurately, comprehensively, and quickly identify large cohorts of insomnia patients.

## Introduction

Sleep-related complaints are second only to complaints of pain as a reason to seek medical attention^1^. Characterized by difficulty falling asleep, staying asleep, or waking unrefreshed, insomnia has a strong impact on the daily lives of affected individuals. In the United States, insomnia is associated with 252.7 million days of lost work per year and an annual cost of $63.2 billion^2^.

Insomnia has been described as an underdiagnosed and undertreated disease in multiple studies^3–5^ and its prevalence has been estimated between 10% and 40%, depending on the definition of insomnia used^6^. These prevalence estimates suggest a potentially high medical burden of insomnia and associated conditions, but existing data are typically from smaller studies in selected populations^7–9^. Furthermore, insomnia studies are insufficient in number, and limited evidence exists for managing more complex insomnia patients and for studying the long-term effects of insomnia treatments^10^. Robust studies of insomnia require long durations and considerable population sizes to detect sufficient outcomes for analysis, and the low frequency of insomnia diagnosis codes in longitudinal databases likely underestimates insomnia’s actual prevalence^11–13^. Therefore, there is a pressing need to develop large cohorts of patients with insomnia that are more thoroughly inclusive and that can be assessed over extended durations.

The capabilities of advances in data analytics with the large volume of data available in electronic medical records (EMRs) allow the use of highly accurate mechanisms to process large collections of clinical notes and enable researchers to conduct a variety of analyses that can add to insights gained from traditional cohorts^14–20^. Specifically, the development of classification algorithms that combine coded data, such as prescriptions or diagnosis codes (structured data), with narrative, textual data, such as physician narrative notes (unstructured data), has been shown to increase the accuracy of identifying patient cohorts with specific diseases^21^.

Our analysis of EMR data showed that documentation of insomnia symptoms in the EMR is rarely detailed enough to use formal case definitions of insomnia such as those from the *Diagnostic and Statistical Manual of Mental Disorders (DSM-V)*^22^. Therefore, we used a more empirical approach to identify insomnia patients. This definition includes criteria such as a physician-documented diagnosis of insomnia (whether via a billing code or a text note), electronic prescriptions for insomnia medications (those indicated only for insomnia), or physician documentation of sleep issues consistent with insomnia (for brevity, we used the term “physician-documented insomnia” to describe this empirical definition). Using a physician-documented insomnia definition allows for the identification of insomnia patients in the EMR who had sleep features not documented in a formal *DSM-V* diagnostic manner.

The objective of our study was to develop a classification algorithm to identify patients with physician-documented insomnia, even when no indication was found in their traditional structured records (such as coded insomnia). We evaluated whether a classification methodology that incorporates clinical narrative notes could be superior to one that solely relies on billing codes. We hypothesized that an EMR text-based algorithm approach allows for more accurate identification of a large number of patients with physician-documented insomnia, especially in comparison to traditional billing-code-based classification schemes. Our study emphasizes the critical need to develop more efficient methods to classify patients with insomnia.

## Results

### Identifying a Physician-Documented Insomnia Cohort using EMR Data

**Figure 1:**
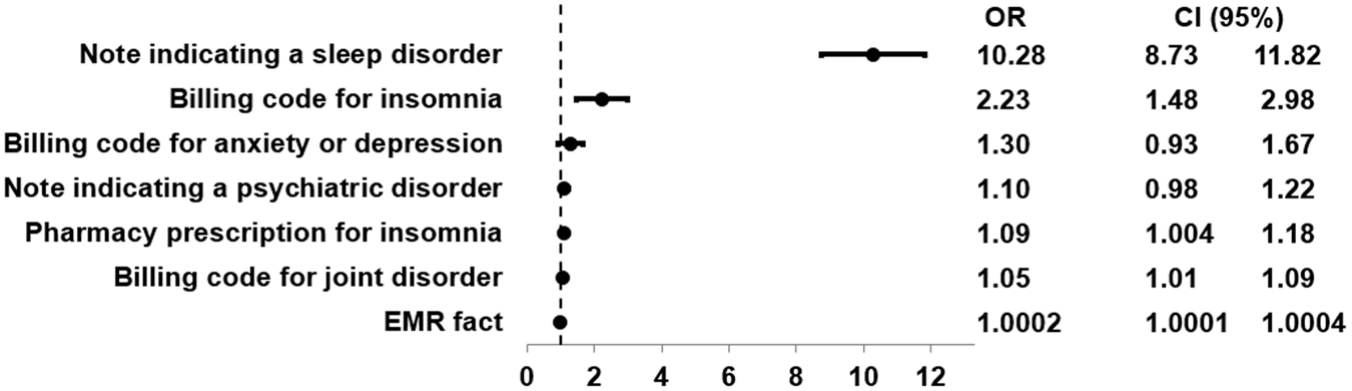
Variables selected for physician-documented insomnia algorithm. OR = odds ratio; CI = confidence interval.

**Figure 2:**
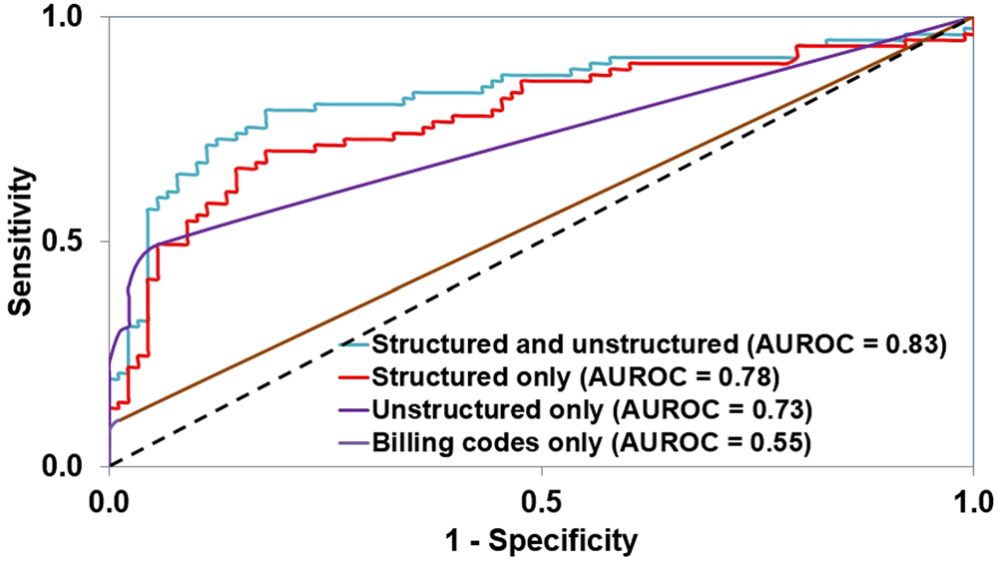
AUROCs of the algorithms for physician-documented insomnia using varying combinations of structured and unstructured (narrative) data. AUROC = area under the receiver operating characteristic curve.

Applying penalized logistic regression to a training set of patients manually annotated for their physician-documented insomnia status led to the selection of seven variables in the final algorithm (Fig. 1). Structured variables in the algorithm, for instance, included the number of ICD-9 codes for insomnia and the number of prescriptions for sleep-related medications. Unstructured variables included the number of narrative note mentions of sleep difficulties as well as the number of narrative note mentions of psychiatric disorders. An additional structured covariate called “# of EMR facts” included the total number of data entries associated with the patient, including, for example, medications, laboratory measurements, notes, comorbidities, and office and emergency room visits. This covariate was able to estimate the patient’s degree of utilization of the care system. The rationale was that patients with a larger number of data entries tend to be sicker than others and thus associated with a higher level of utilization. The algorithm had an area under the receiver operating characteristic curve (AUROC) = 0.83 [95% CI, 0.76–0.90] based on a manual chart review (Fig. 2). Applying this algorithm to the entire insomnia candidate datamart led to a cohort of 36,810 physician-documented insomnia patients (associated with our chosen specificity threshold of 97%).

1 $$\begin{array}{c}{\rm{L}}=-1.927027677\\ \,\,\,\,+\,2.329738590\ast [\#\,{\rm{notes}}\,{\rm{indicating}}\,{\rm{a}}\,{\rm{sleep}}\,{\rm{disorder}}]\\ \,\,\,\,+\,0.802462562\ast [\#\,{\rm{billing}}\,{\rm{codes}}\,{\rm{for}}\,{\rm{insomnia}}]\\ \,\,\,\,+\,0.264231683\ast [\#\,{\rm{billing}}\,{\rm{codes}}\,{\rm{for}}\,{\rm{anxiety}}\,{\rm{or}}\,{\rm{depression}}]\\ \,\,\,\,+\,0.098835169\ast [\#\,{\rm{notes}}\,{\rm{indicating}}\,{\rm{a}}\,{\rm{psychiatric}}\,{\rm{disorder}}]\\ \,\,\,\,+\,0.086364249\ast [\#\,\text{pharmacy}\,{\rm{prescriptions}}\,{\rm{for}}\,{\rm{insomnia}}]\\ \,\,\,\,+\,0.048271004\ast [\#\,\text{billing}\,{\rm{codes}}\,{\rm{for}}\,{\rm{joint}}\,{\rm{disorder}}]\\ \,\,\,\,+\,0.000231521\ast [\#\,{\rm{of}}\,{\rm{EMR}}\,{\rm{facts}}]\end{array}$$

2 $$p(insomnia)=\frac{\exp ({\rm{L}})}{1+\exp ({\rm{L}})}$$

The classification used a logistic regression algorithm that included both structured and unstructured variables. Of note, only 6,159 of 36,810 patients with physician-documented insomnia had an insomnia-related billing code in their EMR history (17%). We constructed analogous logic regression algorithms with different combinations of data subtypes to explore the effect of different data types on algorithm performance. In contrast to the AUROC of 0.83 [95% CI, 0.76–0.90] for our algorithm using both unstructured and structured variables, the AUROC was 0.78 [95% CI, 0.70–0.85] for an algorithm using structured data only, 0.73 [95% CI, 0.67–0.79] using unstructured data only, and only 0.55 [95% CI, 0.51–0.58] using ICD-9 codes only (Fig. 2). Based on a manual chart review, the positive predictive value (PPV) was 0.81 (see Supplementary Table 4). The similar values of AUROCs in the derivation and validation sets in the combined structured and unstructured scenario support the assumption that no overfitting occurred (0.85 [Standard Error: 0.0036] vs. 0.83).

### Physician-Documented Insomnia Cohort Characteristics

Patient characteristics for our cohort are presented in Table 1. In particular, the mean age in our cohort was 62.0 years with 59.4% females. Our cohort was multiethnic, with Caucasian (72.9%), African American (10.4%), Hispanic (10.8%), and Asian (2.0%) patients. The average duration of follow up (time between the first encounter and either the last encounter or death) was 14.1 years; 76.6% of the patients had 10 or more years of follow up.

**Table 1:** Characteristics of physician-documented insomnia cohort. The top 20 conditions of prevalence are shown.

Variable and category	Overall (n = 36,810)
**Age (years); Mean (Standard deviation)**	62.0 (16.3)
**Gender (%)**	
Male	40.6
Female	59.4
**Ethnicity (%)**	
Caucasian	72.9
African American	10.4
Asian	2.0
Hispanic	10.8
Other	1.1
Unknown	2.8
**Marital Status (%)**	
Married or partner	44.4
Other	53.5
Unknown	2.1
**Insurance Type (%); Patients can have more than one type of insurance**.	
Medicaid	6.9
Medicare	56.3
Other	99.4
Body mass index (kg/m^2^); Mean (Standard Deviation)	30 (8.2)
**Smoking Status (%)**	
Current	16.9
Past	23.7
Never	50.3
Unknown	9.1
**Comorbidities; Prevalence (%)**	
Joint disorder	79.3
Hypertension	75.4
Disorders of lipid metabolism	66.5
Diabetes (either type I or II)	55.9
Gastrointestinal disorder	53.0
Anxiety or depression	46.9
Psychiatric disorder	38.4
Pneumonia	37.1
Obesity	34.2
Congestive heart failure	32.7
Coronary artery disease	27.9
Asthma	23.9
Chronic obstructive pulmonary disease	23.5
Cerebrovascular disease	22.9
Atrial fibrillation/Atrial flutter	21.8
Cancer	21.8
Peripheral vascular disease	19.7
Osteoporosis	18.1
Chronic kidney disease/end stage renal disease	16.3
Renal failure	12.3

Joint disorders, hypertension, and disorders of lipid metabolism and diabetes (type 1 or type 2) were the highest prevalent comorbidities, at 79.3%, 75.4%, 66.5%, and 55.9%, respectively. A high proportion of patients suffered from anxiety/depression (46.9%) or a psychiatric disorder (38.4%), and 1.9% of patients suffered from Alzheimer’s disease (including the broader definition for dementia). Several highly prevalent chronic conditions were also highly represented in this cohort, including obesity (34.5%), congestive heart failure (32.7%), coronary artery disease (27.9%), asthma (23.9%), and chronic obstructive pulmonary disease (23.5%).

## Discussion

Sleep disorders are commonly assessed using standardized survey instruments, including the Insomnia Severity Index^23–25^ and the Pittsburgh Sleep Quality Index^26–28^. However, these questionnaires are laborious to administer, rely on patient recall, and are often administered to narrowly define patient populations (e.g.^2,29,30^). In contrast, the development of classification algorithms that rely on EMR data may serve as efficient mechanisms to accurately assess sleep disorders at both the individual and population levels.

Our study describes the development of an algorithm that was used to define a large, longitudinal EMR cohort of patients with a high likelihood for physician-documented insomnia. These patients had an average follow up of 14.1 years, and 76.6% of them had greater than or equal to 10 years of follow-up data, highlighting the breadth of data made available by this algorithm-based cohort identification approach. To our knowledge, our cohort is the largest cohort of insomnia patients (n = 36,810).

As a reassuring validation of our approach, our cohort’s female:male predominance of 1.5 among physician-documented insomnia patients was very similar to the female:male ratio of 1.4 reported in an insomnia meta-analysis^31^. Additionally, the identified insomnia patients had a high prevalence of insomnia-associated diseases, such as psychiatric diseases^32–40^, joint disorders^41–45^, diabetes^46,47^, and stroke/cerebrovascular diseases^48–50^.

Our algorithm demonstrated superiority in identifying insomnia patients over using billing codes alone, which is in line with previous text-inclusive algorithm results for other diseases^21^. Our algorithm performed best when it combined classic structured elements, like billing codes and medication prescriptions, with unstructured data from narrative notes (AUROC = 0.83 [95% CI, 0.76–0.90]). Among our physician-documented insomnia patients, only 17% had one or more billing codes for insomnia, and the AUROC for identification using billing data alone was 0.55 [95% CI, 0.51–0.58]. The markedly lower AUROC with a billing-code-only identification methodology demonstrates the inferiority of this traditional classification scheme and highlights the benefits that a note-incorporating algorithm-based identification can provide. Furthermore, because only 17% of the physician-documented insomnia patients had billing codes for insomnia, this suggests that studies based on billing codes alone may fail to identify a large proportion of insomnia patients. Relying on inaccurate identification of insomnia patients may result in biased experiments that are not representative of the general insomnia population.

Interestingly, even an algorithm incorporating only unstructured data had an AUROC higher than that of a solely billing-code-based identification, validating the effectiveness of features indicating a sleep disorder found in clinical notes. Our algorithm demonstrates the need to increase the use of the large amount of data on insomnia documented in EMR text. This also points to the potential of analyzing narrative text as a powerful new data source to understand insomnia. Extracting disease concepts from clinical narrative notes may more accurately characterize an individual’s health status, especially for patient symptoms or conditions that are not the principal reason for the physician visit (and thus may be less likely to be coded for billing).

Notably, a simplified version of our algorithm, one that considers only the unstructured sleep disorder variable (and ignores the other six variables) can quickly identify patients with insomnia. For instance, per Equations 1 and 2, when a patient has one note indicating a sleep disorder, his or her insomnia probability equals 0.60. When the patient has two notes indicating a sleep disorder, his or her insomnia probability equals 0.94, exceeding the 0.828 threshold and classifying the patient as having insomnia. In another scenario, even when no notes are available, it is possible to use our algorithm based on insomnia billing codes alone. To exceed the insomnia threshold in such a scenario, a patient needs 5 or more insomnia billing codes, having an insomnia probability of 0.89. On the one hand, using simplified versions of our algorithm may not be as accurate as using it with all seven variables. On the other hand, this will allow a rapid initial assessment to estimate the number of patients with insomnia in a given EMR repository, even if it does not contain notes.

EMR documentation did not allow for thorough identification of patients based on traditional *DSM-V* criteria. We believe this enhances our study’s relevance to clinical practice, because our empiric definition (which includes the ability to mine the content of physician notes) reflects the reality of how insomnia is discussed in patient–doctor interactions. It also suggests that traditional studies based on *DSM-V* criteria may be significantly underusing a large volume of the patient health information available in EMR notes and that new methods for classifying insomnia for research purposes are needed.

Although our study describes analyses of retrospective medical databases, our proposed algorithm can be used to identify patients who are at a high-risk of insomnia in real time and thus may inform therapeutic decision-making before the patient has actually developed the condition. In a desirable scenario, our algorithm can calculate the risk of insomnia automatically as an integrated component of an EMR system; the clinician would see a risk score (probability) or a risk rank (low, medium, high) associated with the patient, and this can be used to guide inpatient and outpatient monitoring strategies.

Although extended time frames of EMR data were available for patients in our identified cohort, it is likely that this EMR data was incomplete because various health records may have been stored in other databases not available to us. This is an important limitation of our study. Further, despite the large number of patients in our cohort, our population was derived from urban tertiary-referral hospitals and thus may not be representative of patients seen in other health care contexts. Also, given the integration of prescription and comorbidity data into our algorithm, further studies using this cohort should apply matching methodologies to account for the level of utilization of the health system for insomnia cases vs. controls.

Another limitation of our study is algorithmic. On the one hand, the adaptive least absolute shrinkage and selection operator (LASSO) method identified seven variables and left out potential variables that may also be correlated with insomnia vs. lack of insomnia. Feature selection algorithms are known to be blind to the clinical importance of variables, and when highly correlated variables are identified, the algorithm randomly selects one. Adaptive LASSO has been highly effective in variable selection for the prediction of outcomes related to liver disease^51^, and we thus chose it for our study as well. Additional feature selection algorithms are available, including those based on supervised and unsupervised approaches. Each such algorithm has advantages and disadvantages regarding its ability to identify the most efficient covariates associated with a certain outcome^52,53^.

We do not propose our algorithm as a complete replacement for gold-standard methods for identifying patients with insomnia. However, we do demonstrate a method capable of quickly identifying a large number of patients with insomnia that can be used to find associations traditional studies of smaller scale and shorter duration cannot. We also demonstrate the inferiority of traditional billing code-based classification schemes. Because our algorithm allows for a large-scale and quick approach to insomnia classification, findings resulting from the identified patients can be used to guide hypothesis generation in insomnia research.

In conclusion, EMR approaches may prove to be powerful complementary methods to study the impact of insomnia in large populations, especially if the breadth and depth of knowledge available in clinical notes is used to its fullest potential. We demonstrated a physician-documented insomnia classification algorithm that outperforms billing codes in identifying of physician-documented insomnia and highlighted the importance of finding new approaches for classifying insomnia. Using our clinical note-based approach, we identified the largest insomnia cohort to date, which can be used to conduct large-scale data analyses of this highly prevalent and important medical condition. As a subsequent step, we plan to apply our algorithm on additional populations to further explore the characteristics of patients suffering from insomnia compared to the general population as a whole.

## Methods

### Study Population

**Figure 3:**
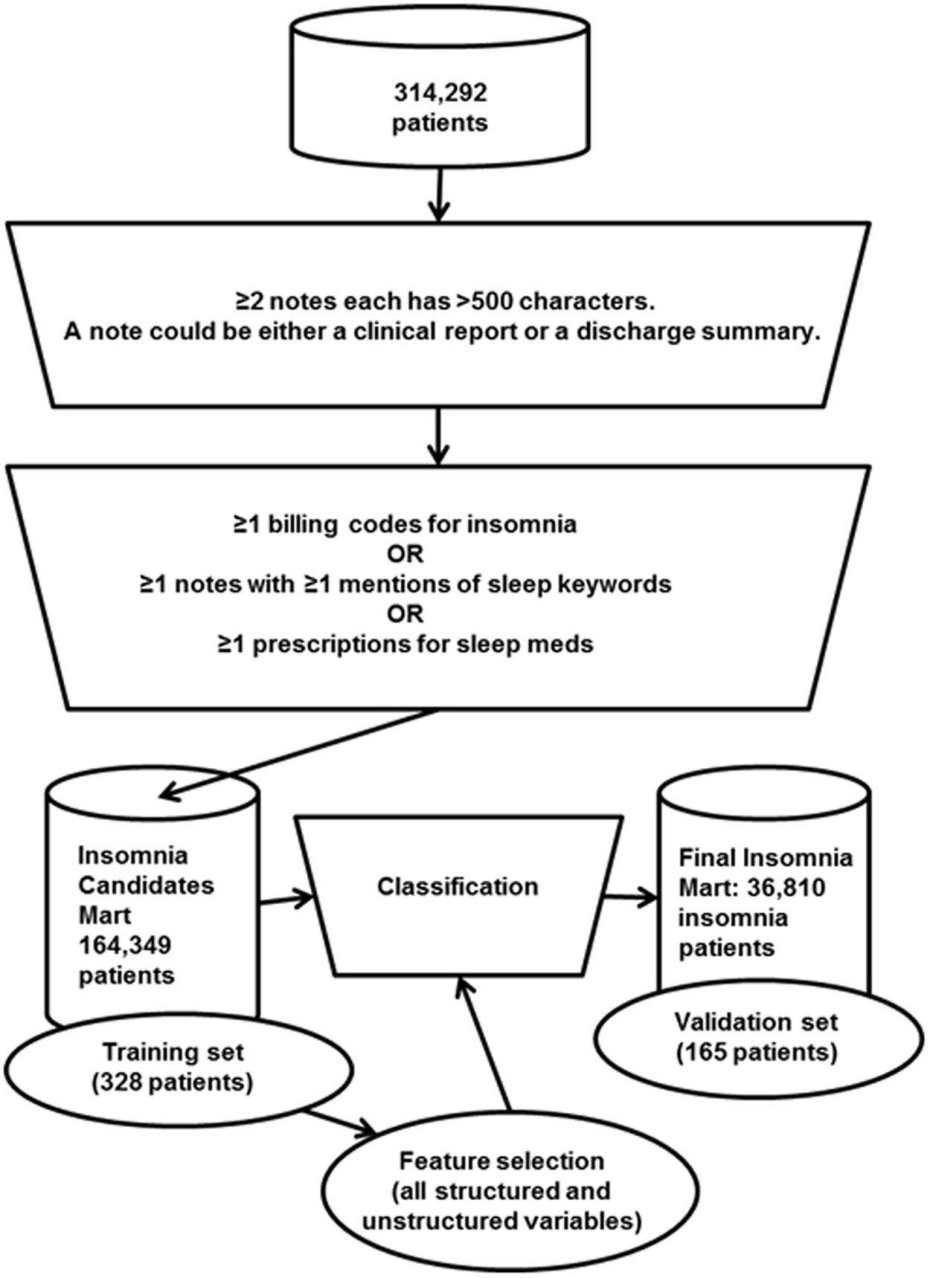
Insomnia algorithm and cohort development. A total of 600 patients were manually labeled: 230 patients had insomnia, 270 patients did not have insomnia, and 100 patients did not have a clear insomnia status. The 500 patients with known insomnia status were used to develop the algorithm; 7 of these 500 patients were excluded because their age was below 18 (date of death or date of the end of the study). Additionally, two-thirds of these (328) served as a training set, while the rest (165) served as a validation set.

We analyzed a previously defined dataset of 314,292 patients from Massachusetts General Hospital and Brigham and Women’s Hospital who received care between 1992 and 2010^54,55^. Patients in this cohort were at increased risk for metabolic syndrome—they had at least one type 2 diabetes mellitus (T2DM) diagnosis code, a T2DM medication, an Hemoglobin A1c (HbA1c) level ≥6.5 percent, or plasma glucose ≥200 mg/dl. From the 314,292 patients, we first created an intermediate dataset of patients (our insomnia candidate mart), based on insomnia-related billing codes, insomnia-related medication prescriptions, and narrative note mentions of sleep-related keywords. Criteria for inclusion in this intermediate cohort included presence of an insomnia-related billing code (307.41, 307.42, 327.00, 327.01, 327.02, 327.09, 780.52) or a sleep-related medication. Additionally, our initial inclusion criteria included patients who had at least one note with a mention of either “sleep” or “insomnia.” The definition identified a broad set of patients who truly had documentation of sleep disorder as well as patients who did not suffer from any sleep difficulties (e.g., “patient sleeps well.”) Only patients with at least two notes of 500 characters or more were included. A total of 164,349 patients made up the insomnia candidate mart (Fig. 3).

A medical student performed a physician-supervised manual chart review to assess the feasibility of using a *DSM-V*-based manual classification scheme. Based on a manual chart review of approximately 50 random insomnia patients, details such as the frequency of symptoms per week or the duration of symptoms, which would be necessary for a *DSM-V* diagnosis, were not documented for the vast majority of patients. These observations from our data are consistent with a previous report^56^. As a result, criteria such as *DSM-V* would be impractical to classify the insomnia status of patients in the EMR. Therefore, we supplemented *DSM-V* with more empirical criteria, considering the criteria presented in Supplementary Table 1. For brevity, we used the term “physician-documented insomnia” to describe this empirical definition. All manual chart reviews were performed by RA under the guidance of SYS.

A total of 600 randomly selected patients from the insomnia candidate datamart were subjected to manual EMR chart review. This chart review was physician supervised and done by a medical student to ensure consistency of labeling. Patients were classified as “physician-documented insomnia” (230), “no evidence of physician-documented insomnia” (270), or “undetermined” (100). Among the 500 patients with known insomnia status (positive or negative), seven were excluded because they were younger than 18 either at their date of death or at the date of the end of the study. Two-thirds of the patients (328) served as a training set and one-third (165) were designated as a validation set for algorithm development (Fig. 3).

Because EMR documentation did not support use of formal case definitions for insomnia (see Results), our insomnia case definition supplemented formal insomnia diagnostic criteria (*DSM-V*) with additional empirical criteria. These empirical criteria included a physician-documented diagnosis of insomnia, or a physician documenting sleep-related difficulty consistent with insomnia on different occasions throughout the patient’s life-time. Additionally, the criteria included a physician prescribing a medication that was indicated only for insomnia (Ambien/Zolpidem, Ambien CR/Zolpidem CR, Lunesta/Eszopiclone, Restoril/Temazepam, Sonata/Zaleplon, Dalmane/Flurazepam, and ProSom/Eurodin/Estazolam). We selected these medications because they are known primarily for treatment of troubled sleeping (as opposed to medications primarily used to treat other conditions, such as depression or anxiety, whose second use can also help with insomnia, such as Desyrel/trazodone). Furthermore, we considered only medications that were available during our study period (between 1992 and 2010) and not newer ones irrelevant to our study (e.g., Belsomra/Suvorexant). The presence of any one of the classification criteria was sufficient for an empiric classification of a patient as physician-documented insomnia (Supplementary Table 1).

### Covariate Definition

Structured variables were defined using ICD9 codes and current procedural terminology codes and included a broad range of comorbidities (Supplementary Table 2). To extract unstructured variables, we used text nailing (TN), a text-processing method that members of our group developed. TN is based on using an interactive human-in-the-loop mechanism to identify nonnegated clinical and behavioral descriptors, and it was proved to be highly accurate compared to traditional machine learning algorithms^57,58^. One advantage of TN is that it is not sensitive to negations; thus, it can identify expressions that truly indicate the existence of a certain condition or behavior. As in our recent study published in *Scientific Reports*^59^, we used the top 10 expressions with the highest prevalence to define a sleep disorder incorporated by our algorithm. The expressions were: “poor sleep,” “has trouble sleep,” “reduced sleep,” “increased sleep,” “decreased sleep,” “excessive sleep,” “fragmented sleep,” “sleeplessness,” “sleep disruption,” and “sleeps poorly.” All the definitions for our unstructured variables can be seen in Supplementary Table 3.

### Classification Modeling

We applied logistic regression with the adaptive LASSO to develop a classification algorithm for physician-documented insomnia. We evaluated different combinations of variable subtypes, including (1) ICD-9 codes related to insomnia, (2) structured variables (e.g., ICD-9 codes of variety of comorbidities, prescriptions, demographics), (3) only narrative (unstructured) variables, and (4) a combination of structured and unstructured variables. We used these four combinations to assess the optimal algorithm for identifying patients with physician-documented insomnia. We chose the adaptive LASSO over other feature selection methods because it is considered an efficient algorithm for parsimoniously ranking variables in clinical predictive modeling^60,61^.

We used a specificity threshold of 97% for classifying physician-documented insomnia patients, which corresponded to a probability threshold (for physician-documented insomnia) of 0.828. To calculate 95% confidence intervals, we applied the bootstrap procedure with 1,000 replicates. We calculated the AUROCs to measure the model’s accuracy in the validation set. Additionally, we evaluated for overfitting by comparing the AUROC in the validation set to an average AUROC value for 100 permutations of randomly selected sub-derivation and sub-validation sets in the training set. We then applied our algorithm to the 164,349 patients in the insomnia candidate datamart using Equations 1 and 2 to calculate the probability of having physician-documented insomnia for each patient.

### Validation

To validate our algorithm, we randomly selected a distinct set of 300 patients from the intermediate dataset of 164,349 individuals. Manual review identified 200 patients who could be labeled as having insomnia or not; of these, 88 patients had insomnia, and 112 patients did not have insomnia. We excluded two of the patients because they were younger than 18 either at their date of death or at the date of the end of the study. A performance summary to assess the algorithm performance in the remaining 198 patients is presented in Supplementary Table 4. Based on this, the final PPV of our algorithm was calculated.

The institutional review board of Partners HealthCare approved this study and all its methods, including the EMR cohort assembly, data extraction, and analyses. The present project was reviewed and approved with a waiver of informed consent from the institutional review board at Partners HealthCare. Furthermore, we confirm that all methods were performed in accordance with the relevant guidelines and regulations of Scientific Reports. Data contain potentially identifying information and may not be shared publicly. Deidentified data may be requested from The Partners Human Research Committee, the Institutional Review Board of Partners HealthCare (Address: 399 Revolution Drive, Suite # 710, Somerville MA, 02145, USA, Telephone: 857-282-1900).

## Electronic supplementary material

SUPPLEMENTARY
