# Supplementary material for: Development of an Algorithm to Identify Patients with Physician-Documented Insomnia
Source: Sci Rep. 2018 May 18;8:7862. doi: 10.1038/s41598-018-25312-z (PMC5959894; doi:10.1038/s41598-018-25312-z)
Supplement: Supplementary file 1 — SUPPLEMENTARY [file 41598_2018_25312_MOESM1_ESM.docx]

**Development of an Algorithm to Identify Patients with Physician-Documented Insomnia**

**Uri Kartoun PhD^1,2,8^, Rahul Aggarwal BA^1,2^, Andrew L Beam PhD^2,3^, Jennifer K Pai ScD MHS^4^, Arnaub K Chatterjee MHA MPA^4,5^, Timothy P Fitzgerald PhD^6^,** **Isaac S Kohane MD PhD^2,3^, Stanley Y Shaw MD PhD^1,2,7,*^**

1. Center for Systems Biology; Center for Assessment Technology & Continuous Health (CATCH),

Massachusetts General Hospital, Boston, MA, USA.

2. Harvard Medical School, Boston, MA, USA.

3. Department of Biomedical Informatics, Harvard Medical School, Boston, MA, USA.

4. Merck & Co., Inc., Boston, MA, USA.

5. Current address: McKinsey & Company, Boston, MA, USA.

6. Merck & Co., Inc., West Point, PA, USA.

7. Current address: One Brave Idea, Division of Cardiovascular Medicine, Brigham and Women’s Hospital, Boston, MA, USA.

8. Current address: Center for Computational Health, IBM Research, Cambridge, MA, USA.

*** Corresponding author**

E-mail: [stanley_shaw@hms.harvard.edu](mailto:stanley_shaw@hms.harvard.edu)

**Supplementary Tables**

**Supplementary Table 1. Criteria used to identify insomnia by a chart review**

| Physician states patient has insomnia. |
| --- |
| Physician gives medication with indication for insomnia. |
| Patient on medication that is solely used for insomnia management. |
| 3 or more mentions of sleep problems in notes, that are not localized to one night and not defined by another sleep disorder. |
| Waking up multiple times at night and the presence of daytime impairment, not defined by sleep apnea, and not localized to one night. |
| 2 different medications with indication for sleep problems and 1 mention of sleep difficulty that is not localized to one night. |
| Explicitly defines sleep as a primary issue and is prescribed to a sleep medication. |
| 2 mentions of sleep problems with daytime impairment present that is not localized to one night. |
| Patient waking up too early (defined as 4am or before for someone not on a nocturnal schedule), not falling back asleep, and these problems are described as occurring often. |
| Mention of difficulty falling asleep, maintaining sleep, or waking up early of greater than 30 minutes duration with daytime impairment mentioned in the same note and the occurrences are not isolated to one night. |

**Supplementary Table 2. Diagnosis, procedure, and CPT codes used to define comorbidities**

| **Comorbidity** | **ICD9 Diagnosis Codes** | **ICD9 Procedure Codes** | **CPT Codes** |
| --- | --- | --- | --- |
| **Alzheimer’s disease / dementia** | 331.0, 294.1  Dementia: 290, 294.1 | - | - |
| **Anxiety, depression, dissociative disorders, and somatoform disorders** | 296.2, 296.3, 300.x | - | - |
| **Asthma** | 493.x | - | - |
| **Atrial fibrillation /**  **Atrial flutter** | 427.31, 427.3, 427.32 | 37.33, 37.34 | 93653 to 93657, 33254 to 33259, 33265 to 33266 |
| **Cancer** | Breast cancer: 174.x  Colon cancer: 153.x  Esophageal cancer: 150.x  Pancreatic cancer: 157.x  Prostate cancer: 185.x  Renal cancer: 189.x  Thyroid cancer: 193.x  Gall bladder cancer: 156.x  Uterine cancer: 179.x  Cervical cancer: 180.x  Lung cancer: 162.x | - | - |
| **Cerebrovascular disease** | 430.x, 431.x, 432.x, 433.x, 436 | - | - |
| **Chronic kidney disease / end stage renal disease** | 585.1, 585.2, 585.3, 585.4, 585.5, 585.6, 585.9 | - | - |
| **Cirrhosis** | 571.2, 571.5, 571.6 | - | - |
| **Congestive heart failure** | 428.x |  |  |
| **COPD** | 491.x, 492.x, 496 | - | - |
| **Coronary artery disease (ischemic heart disease / myocardial infarction)** | 410.x, 411.x, 412.x, 413.x, 414.x | 36.x, 00.66 | 92995, 92996, 92982, 92984, 92980, 92981, 33510 to 33545 |
| **Diabetes** | 250.x | - | - |
| **Disorders of lipid metabolism (dyslipidemia, hyperlipidemia, and related others)** | 272, 272.0, 272.1, 272.2, 272.3, 272.4, 272.5, 272.6, 272.7, 272.8, 272.9 | - | - |
| **Gastrointestinal disorder** | Duodenal ulcer: 532.x  Esophageal reflux: 530.81  Esophagitis: 530.10  Gastric ulcer: 531.x  Gastritis and Duodenitis: 535.x  Gastrojejunal ulcer: 534.x  Peptic ulcer: 533.x  Ulcer of esophagus: 530.20 | - | - |
| **Hypertension** | 401.x, 997.91 | - | - |
| **Joint disorder** | Crystal arthropathies: 712.x  Rheumatoid arthritis and other inflammatory Polyarthropathies: 714.x  Dorsopathies: 720.x, 721.x, 722.x, 723.x, 724.x  Internal derangement of knee: 717.x  Osteoarthrosis and allied disorder: 715.x  Psoriatic arthropathy: 696.0 | - | - |
| **NAFLD** | 571.8, 571.9 | - | - |
| **Non-viral hepatitis** | 571.1, 571.4x | - | - |
| **Obesity** | 278, 278.0, 278.00, 278.01, 278.02 | - | - |
| **Osteoporosis** | 733.0x | - | - |
| **Peripheral vascular disease** | 443.9, 785.4, 250.7 443.81, 440.21, 440.22, 440.23 | 84.1x, 00.55, 39.90, 00.60, 39.22, 39.24, 39.25, 39.26, 39.50, 38.13, 38.18, 00.40 to 00.43, 00.46 to 00.48 | 37220, 37222, 37224, 37228, 37232, 37205, 37221, 37223, 37226, 37230, 37234, 27590 to 27598, 28800, 28805, 28810, 28820, 28825, 27880, 27881, 27882, 27884, 27886, 28888, 27889, 27290, 27295 |
| **Pneumonia** | 480.x, 481.x, 482.x, 483.x, 484.x, 485.x, 486.x, 487.0, 488.01, 488.11 | - | - |
| **Psychiatric disorder** | 294.0, 294.8, 294.9, 295.x, 296.x, 297.x 298.x (excluding 296.2, 296.3) | - | - |
| **Renal failure** | 250.4, 585, 583.81, 581.81 | 00.91, 00.92, 00.93, 39.95, 54.98 | 90935, 90937, 90945, 90947, 90999, 50360, 50365 |
| **Stroke** | 434.91 | - | - |
| **Viral hepatitis** | 070.x | - | - |

**Supplementary Table 3. Expressions used to define unstructured variables**

| **Variable** | **Expression(s)** |
| --- | --- |
| **Sleep disorder** | poor sleep  has trouble sleep  increased sleep  decreased sleep  reduced sleep  excessive sleep  fragmented sleep  sleeplessness  sleep disruption  sleeps poorly |
| **Alcohol use** | has a history of alcohol  problems alcohol abuse  used alcohol yes  history of depression and alcohol abuse  alcohol use status abuse  hx of depression and alcohol abuse  excessive alcohol intake  he has a history of alcohol abuse  past alcohol abuse  alcohol abuse recovering  he also has a history of alcohol abuse  alcohol abuse in recovery  he also has a history of chronic alcohol abuse  is a known alcoholic  is known alcoholic  has a remote history of alcohol abuse  with history of alcohol abuse  has history of alcohol  alcohol abuse heavy drinking  has a ho alcohol abuse  admits to prior history of alcohol abuse  he has a history of chronic alcohol abuse  history is notable for heavy alcohol abuse  is known as alcoholic  has ho alcohol abuse  has ho of alcohol abuse  has history of alcohol abuse  ho of depression and alcohol abuse  history is notable for alcohol abuse  history is notable for heavy alcohol abuse  alcohol ho of etoh abuse |
| **Psychiatric disorder** | bipolar disorder  mania depression  manic depression  bipolar depression  manic depressive  schizophren  mood swings  mood disorder  psychoses  psychotic  paranoid  paranoia  delusion  delusive |

**Supplementary Table 4. Classification results.** The numbers represent individual patients. Yes = patient has insomnia; No = patient does not have insomnia.

|  | | Chart review | |
| --- | --- | --- | --- |
|  |  | Yes | No |
| Algorithm  Classification | Yes | 42 | 10 |
|  | No | 45 | 101 |
